# ASO Author Reflections: Conventional Versus Videoscopic Inguinal Lymphadenectomy in Surgical Oncology—Reflections on the Surgical Challenges in Current Practice

**DOI:** 10.1245/s10434-026-19501-8

**Published:** 2026-04-13

**Authors:** N. Servaas

**Affiliations:** Zuyderland Medical Centre, Sittard-Geleen, The Netherlands

## Past

Inguinal lymphadenectomy (IL) remains an important surgical procedure in the treatment of various stage III malignancies. IL is notorious for postoperative complications, such as surgical site infections, seroma formation, skin flap necrosis, wound breakdown, and lymphedema.^1–3^ Long-term morbidity after IL is common and surgical reinterventions are often required to treat complications. Over recent years, studies such as the MSLT-II and DeCog-SLT have reduced the indication for routine lymphadenectomies in stage III melanoma.^4^ However, IL remains crucial in specific cases with stage III disease. This article reflects on our retrospective cohort study on conventional versus videoscopic inguinal lymphadenectomy and includes a reflection on the challenges in current clinical practice.^5^

## Present

Innovations in surgical practice with the aim to reduce complications are crucial for improving patient-related outcomes. Our study shows that videoscopic IL reduced complication rates, the severity of complications, and early surgical reinterventions when compared with the conventional approach. Seroma formation and lymphedema are common, while preventive strategies have demonstrated a limited effect. Postoperative seroma formation and lymphedema remain an important challenge in clinical practice. No adequate solutions have been identified to combat these complications. Although indications for IL have reduced in recent years, IL remains an important procedure in surgical oncology. When performed, videoscopic IL offers a transformative approach which is superior to conventional IL.

## Future

Although systemic treatment is becoming more important in current practice, IL will remain pivotal in specific cases of stage III malignancies. Findings on IL in literature are often limited by their sample size and retrospective design. Future research should focus on innovative strategies to prevent seroma formation and lymphedema. The optimization of drainage protocols could contribute to these outcomes. This article helps guide surgical practice by reducing postoperative wound complications. Additional innovations will further improve the quality of the surgical practice, ultimately reducing the morbidity of IL.

## References

[1] Chang SB, Askew RL, Xing Y, et al. Prospective assessment of postoperative complications and associated costs following inguinal lymph node dissection (ILND) in melanoma patients. *Ann Surg Oncol*. 2010;17(10):2764–72. 10.1245/s10434-010-1026-z. 20336388 10.1245/s10434-010-1026-z PMC2943041

[2] Poos HPAM, Kruijff S, Bastiaannet E, van Ginkel RJ, Hoekstra HJ. Therapeutic groin dissection for melanoma: risk factors for short term morbidity. *Eur J Surg Oncol*. 2009;35(8):877–83. 10.1016/j.ejso.2008.10.012. 19054644 10.1016/j.ejso.2008.10.012

[3] Stuiver MM, Westerduin E, ter Meulen S, Vincent AD, Nieweg OE, Wouters MWJM. Surgical wound complications after groin dissection in melanoma patients—a historical cohort study and risk factor analysis. *Eur J Surg Oncol*. 2014;40(10):1284–90. 10.1016/j.ejso.2014.01.019. 24612654 10.1016/j.ejso.2014.01.019

[4] Bello DM, Faries MB. The landmark series: MSLT-1, MSLT-2 and DeCOG (management of lymph nodes). *Ann Surg Oncol*. 2020;27(1):15–21. 10.1245/s10434-019-07830-w. 31535299 10.1245/s10434-019-07830-w

[5] Servaas N, Aldenhoven L, Spiekerman van Weezelenburg MA, et al. Conventional versus videoscopic inguinal lymphadenectomy in surgical oncology: a retrospective observational cohort study. *Ann Surg Oncol.* 2026; 10.1245/s10434-025-19041-7 10.1245/s10434-025-19041-7 PMC13179235 41591565

